# 4-*tert*-Butyl­pyridinium triiodide–4-*tert*-butyl­pyridine (1/1)

**DOI:** 10.1107/S1600536811001371

**Published:** 2011-01-22

**Authors:** Hongshan He, Andrew G. Sykes

**Affiliations:** a Center for Advanced Photovoltaics, Department of Electrical Engineering and Computer Science, South Dakota State University, Brookings, SD 57007, USA; b Department of Chemistry, University of South Dakota, Vermillion, SD 57069, USA

## Abstract

The title compound, C_9_H_14_N^+^·I_3_^−^·C_9_H_13_N, consists of monoprotonated 4-*tert*-butyl­pyridinium cations and triiodide anions. The triiodide ion has near-symmetric linear geometry, with bond lengths of 2.9105 (4) Å (I—I) and a bond angle of 177.55 (3)° (I—I—I). For this room-temperature structure, the butyl group on the pyridine ring is disordered and has been treated as a rigid rotator, modeled in three separate positions with 1/3, 1/3, 1/3 occupancies. The cations assemble into dimeric forms by way of N—H⋯N hydrogen bonds.

## Related literature

For applications of the 4-*t*-butyl­pyridine and iodide/triiodide system in dye-sensitized solar cells see: Campbell *et al.* (2004 ▶); Lee *et al.* (2010 ▶); Wang *et al.* (2005 ▶). For related structures, see: Fialho *et al.* (1996 ▶); Kochel (2006 ▶).
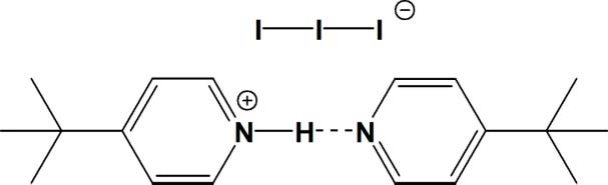


## Experimental

### 

#### Crystal data

C_9_H_14_N^+^·I_3_^−^·C_9_H_13_N
*M* *_r_* = 652.12
Tetragonal, 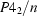

*a* = 11.6862 (4) Å
*c* = 17.1665 (13) Å
*V* = 2344.4 (2) Å^3^
*Z* = 4
Mo *K*α radiation
μ = 4.00 mm^−1^
*T* = 293 K
0.55 × 0.50 × 0.40 mm

#### Data collection

Bruker APEXII CCD area-detector diffractometer
Absorption correction: multi-scan (*SADABS*; Bruker, 2006 ▶) *T*_min_ = 0.217, *T*_max_ = 0.298
23722 measured reflections
2217 independent reflections
1758 reflections with *I* > 2σ(*I*)
*R*_int_ = 0.027

#### Refinement

*R*[*F* ^2^ > 2σ(*F* ^2^)] = 0.037
*wR*(*F* ^2^) = 0.090
*S* = 1.04
2217 reflections
119 parameters
1 restraint
H atoms treated by a mixture of independent and constrained refinement
Δρ_max_ = 0.80 e Å^−3^
Δρ_min_ = −0.77 e Å^−3^

### 

Data collection: *APEX2* (Bruker, 2006 ▶); cell refinement: *SAINT* (Bruker, 2006 ▶); data reduction: *SAINT*; program(s) used to solve structure: *SHELXS97* (Sheldrick, 2008 ▶); program(s) used to refine structure: *SHELXL97* (Sheldrick, 2008 ▶); molecular graphics: *ORTEP-3 for Windows* (Farrugia, 1997 ▶); software used to prepare material for publication: *SHELXTL* (Sheldrick, 2008 ▶), *WinGX* (Farrugia, 1999 ▶) and *publCIF* (Westrip, 2010 ▶).

## Supplementary Material

Crystal structure: contains datablocks global, I. DOI: 10.1107/S1600536811001371/sj5091sup1.cif

Structure factors: contains datablocks I. DOI: 10.1107/S1600536811001371/sj5091Isup2.hkl

Additional supplementary materials:  crystallographic information; 3D view; checkCIF report

## Figures and Tables

**Table 1:** Hydrogen-bond geometry (Å, °)

*D*—H⋯*A*	*D*—H	H⋯*A*	*D*⋯*A*	*D*—H⋯*A*
N1—H99⋯N1^i^	0.90	1.76	2.655 (7)	172

Symmetry code: (i) 
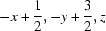
.

**Fig. 1.**
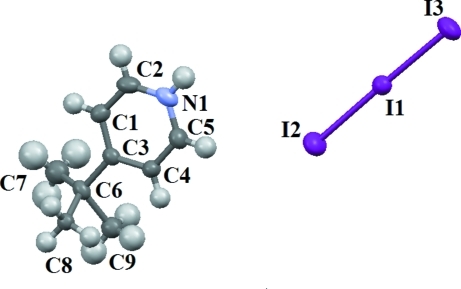
The molecular structure of the title compound showing 30% probability displacement ellipsoids and the atomic numbering.

**Fig. 2.**
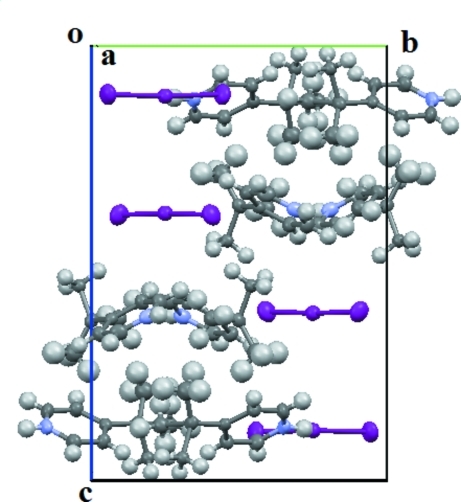
Packing diagram of the title compound, viewed down the a axis.

**Fig. 3.**
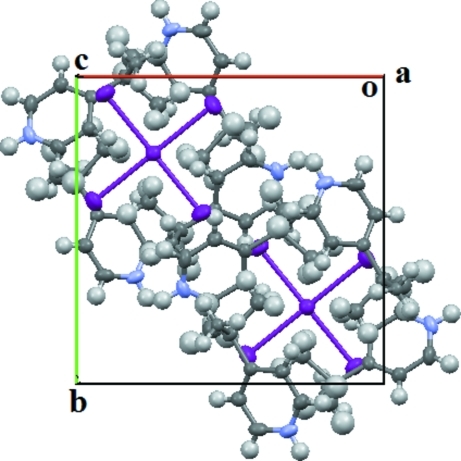
Packing diagram of the title compound, viewed down the c axis.

**Fig. 4.**
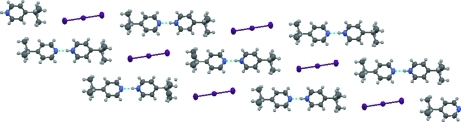
Packing diagram of the title compound showing the hydrogen bonding.

## supplementary crystallographic information

### Comment

4 - *t*-Butylpyridine is usually added into an iodide/triiodide electrolyte solution to enhance the photovoltaic performance of dye-sensitized solar cells. The solution is a mixture of iodide, lithium iodide, 4 - *t*-butylpyridine, and guanidinium thiocyanate (Campbell *et al.*, 2004; Lee *et al.*, 2010). Alternatively, the electrolyte can be prepared by the reaction between 2-hydroxypropionitrile and lithium iodide (Wang *et al.*, 2005). It was proposed that triiodide was produced during the reaction; however, no direct evidence was obtained. Reported here is the structure of the resulting compound.

In the molecule of the title compound, three iodide atoms in triiodide ion are in a linear geometry (Fig. 1). The I1—I2 bond length is 2.9105 (4)Å and the I2—I1—I2 angle is 177.55 (3)°.  The triiodide bond is almost parallel to the pyridyl ring. In each asymmetric unit cell, two pairs of triiodide ions are perpendicular to each other (Fig. 2 and Fig. 3). The cations assemble into dimeric forms by way of N—H···N hydrogen bonds (Fig. 4, Table 1).

### Experimental

2-Hydroxypropionitrile (6.1 g) and lithium iodide (10 g) was added to a flask. The resulting mixture was heated to 120°C in a sealed high pressure tube for 30 minutes. When the temperature decreased to 70°C, silica powder with diameter 25 µm (1.3 g), 4 - *t*-butylpyridine (1.0 g), and ethanol (3 ml) were added. The mixture was stirred by a mechanical stirrer at 50°C for 30 minutes. Red crystals were obtained from the resulting mixture in one month.

### Refinement

The hydrogen that binds to N atom is refined and other hydrogen atoms are geometrically constrained and refined in riding mode as follows: methyl d(C—H) = 0.96 Å, Uĩso~(H) = 1.5U~eq~(C); aromatic d(C—H) = 0.93 Å, Uĩso~(H) = 1.2U~eq~(C). The butyl group on the pyridine ring is disordered and has been treated as a rigid rotator, modeled in three separate positions with 1/3,1/3, and 1/3 occupancies.  All atoms involved have been refined isotropically.

### Crystal data

**Table:**

C_9_H_14_N^+^·I_3_^−^·C_9_H_13_N	*D*_x_ = 1.848 Mg m^−^^3^
*M**_r_* = 652.12	Mo *K*α radiation, λ = 0.71073 Å
Tetragonal, *P*4_2_/*n*	Cell parameters from 9942 reflections
Hall symbol: -P 4bc	θ = 2.4–25.6°
*a* = 11.6862 (4) Å	µ = 4.00 mm^−^^1^
*c* = 17.1665 (13) Å	*T* = 293 K
*V* = 2344.4 (2) Å^3^	Block, red
*Z* = 4	0.55 × 0.50 × 0.40 mm
*F*(000) = 1232	

### Data collection

**Table:**

Bruker APEXII CCD area-detector diffractometer	2217 independent reflections
Radiation source: fine-focus sealed tube	1758 reflections with *I* > 2σ(*I*)
graphite	*R*_int_ = 0.027
φ and ω scans	θ_max_ = 25.6°, θ_min_ = 2.1°
Absorption correction: multi-scan (*SADABS*; Bruker, 2006)	*h* = −14→14
*T*_min_ = 0.217, *T*_max_ = 0.298	*k* = −14→14
23722 measured reflections	*l* = −20→20

### Refinement

**Table:**

Refinement on *F*^2^	Secondary atom site location: difference Fourier map
Least-squares matrix: full	Hydrogen site location: inferred from neighbouring sites
*R*[*F*^2^ > 2σ(*F*^2^)] = 0.037	H atoms treated by a mixture of independent and constrained refinement
*wR*(*F*^2^) = 0.090	*w* = 1/[σ^2^(*F*_o_^2^) + (0.0303*P*)^2^ + 5.5874*P*] where *P* = (*F*_o_^2^ + 2*F*_c_^2^)/3
*S* = 1.04	(Δ/σ)_max_ < 0.001
2217 reflections	Δρ_max_ = 0.80 e Å^−^^3^
119 parameters	Δρ_min_ = −0.77 e Å^−^^3^
1 restraint	Extinction correction: *SHELXL97* (Sheldrick, 2008), Fc^*^=kFc[1+0.001xFc^2^λ^3^/sin(2θ)]^-1/4^
Primary atom site location: structure-invariant direct methods	Extinction coefficient: 0.00208 (15)

### Special details

**Table:**

Geometry. All e.s.d.'s (except the e.s.d. in the dihedral angle between two l.s. planes) are estimated using the full covariance matrix. The cell e.s.d.'s are taken into account individually in the estimation of e.s.d.'s in distances, angles and torsion angles; correlations between e.s.d.'s in cell parameters are only used when they are defined by crystal symmetry. An approximate (isotropic) treatment of cell e.s.d.'s is used for estimating e.s.d.'s involving l.s. planes.
Refinement. Refinement of *F*^2^ against ALL reflections. The weighted *R*-factor *wR* and goodness of fit *S* are based on *F*^2^, conventional *R*-factors *R* are based on *F*, with *F* set to zero for negative *F*^2^. The threshold expression of *F*^2^ > σ(*F*^2^) is used only for calculating *R*-factors(gt) *etc*. and is not relevant to the choice of reflections for refinement. *R*-factors based on *F*^2^ are statistically about twice as large as those based on *F*, and *R*- factors based on ALL data will be even larger.

### Fractional atomic coordinates and isotropic or equivalent isotropic displacement parameters (Å^2^)

**Table:**

	*x*	*y*	*z*	*U*_iso_*/*U*_eq_	Occ. (<1)
C1	0.0501 (4)	0.9717 (5)	0.3368 (3)	0.0641 (12)	
H1	0.0502	1.0414	0.3107	0.077*	
C2	0.1482 (4)	0.9081 (6)	0.3407 (3)	0.0749 (15)	
H2	0.2140	0.9358	0.3169	0.090*	
C3	0.0595 (5)	0.7687 (5)	0.4119 (3)	0.0694 (14)	
H3	0.0625	0.6988	0.4376	0.083*	
C4	−0.0414 (4)	0.8291 (4)	0.4105 (3)	0.0608 (12)	
H4	−0.1055	0.8001	0.4360	0.073*	
C5	−0.0485 (4)	0.9329 (4)	0.3715 (2)	0.0505 (10)	
C6	−0.1613 (4)	0.9989 (4)	0.3681 (3)	0.0590 (12)	
C7	−0.179 (2)	1.059 (2)	0.2943 (13)	0.099 (7)*	0.33
H7A	−0.1554	1.0099	0.2521	0.148*	0.33
H7B	−0.1338	1.1273	0.2937	0.148*	0.33
H7C	−0.2582	1.0775	0.2886	0.148*	0.33
C8	−0.2650 (16)	0.9078 (16)	0.3731 (13)	0.077 (5)*	0.33
H8A	−0.2680	0.8641	0.3258	0.116*	0.33
H8B	−0.3359	0.9479	0.3801	0.116*	0.33
H8C	−0.2526	0.8573	0.4164	0.116*	0.33
C9	−0.1911 (15)	1.0409 (17)	0.4482 (9)	0.051 (4)*	0.33
H9A	−0.1303	1.0882	0.4675	0.076*	0.33
H9B	−0.2017	0.9767	0.4823	0.076*	0.33
H9C	−0.2606	1.0847	0.4459	0.076*	0.33
C7'	−0.144 (2)	1.125 (2)	0.3487 (17)	0.118 (8)*	0.33
H7'1	−0.1136	1.1639	0.3933	0.177*	0.33
H7'2	−0.2159	1.1584	0.3344	0.177*	0.33
H7'3	−0.0912	1.1316	0.3060	0.177*	0.33
C8'	−0.230 (2)	0.953 (2)	0.3061 (13)	0.091 (7)*	0.33
H8'1	−0.3007	0.9947	0.3031	0.136*	0.33
H8'2	−0.2453	0.8739	0.3159	0.136*	0.33
H8'3	−0.1894	0.9609	0.2577	0.136*	0.33
C9'	−0.2236 (18)	0.9924 (19)	0.4480 (12)	0.085 (6)*	0.33
H9'1	−0.1681	0.9934	0.4892	0.127*	0.33
H9'2	−0.2673	0.9230	0.4507	0.127*	0.33
H9'3	−0.2739	1.0569	0.4534	0.127*	0.33
C8"	−0.2550 (14)	0.9235 (14)	0.3301 (12)	0.052 (3)*	0.33
H8"1	−0.3257	0.9653	0.3282	0.078*	0.33
H8"2	−0.2653	0.8553	0.3604	0.078*	0.33
H8"3	−0.2321	0.9033	0.2782	0.078*	0.33
C7"	−0.1486 (14)	1.1071 (15)	0.3079 (11)	0.057 (4)*	0.33
H7"1	−0.0932	1.1602	0.3280	0.085*	0.33
H7"2	−0.2212	1.1449	0.3027	0.085*	0.33
H7"3	−0.1240	1.0798	0.2579	0.085*	0.33
C9"	−0.1668 (19)	1.0791 (19)	0.4363 (13)	0.085 (7)*	0.33
H9"1	−0.0907	1.1022	0.4504	0.127*	0.33
H9"2	−0.2021	1.0410	0.4796	0.127*	0.33
H9"3	−0.2110	1.1452	0.4224	0.127*	0.33
N1	0.1527 (4)	0.8086 (4)	0.3770 (3)	0.0699 (12)	
I1	0.2500	0.2500	0.38499 (3)	0.06646 (18)	
I2	0.05632 (4)	0.40649 (4)	0.38862 (3)	0.1013 (2)	
H99	0.220 (6)	0.772 (11)	0.381 (5)	0.09 (4)*	0.50

### Atomic displacement parameters (Å^2^)

**Table:**

	*U*^11^	*U*^22^	*U*^33^	*U*^12^	*U*^13^	*U*^23^
C1	0.060 (3)	0.068 (3)	0.065 (3)	−0.004 (2)	0.000 (2)	0.009 (2)
C2	0.048 (3)	0.102 (4)	0.074 (4)	−0.002 (3)	0.008 (3)	−0.001 (3)
C3	0.067 (3)	0.068 (3)	0.073 (3)	0.013 (3)	−0.004 (3)	0.008 (3)
C4	0.055 (3)	0.059 (3)	0.068 (3)	0.001 (2)	0.007 (2)	0.007 (2)
C5	0.050 (2)	0.055 (3)	0.047 (2)	0.0031 (19)	−0.0037 (19)	−0.0077 (19)
C6	0.057 (3)	0.060 (3)	0.061 (3)	0.014 (2)	−0.006 (2)	−0.010 (2)
N1	0.053 (3)	0.091 (3)	0.066 (3)	0.020 (2)	−0.005 (2)	−0.011 (2)
I1	0.0663 (3)	0.0676 (3)	0.0656 (3)	0.0000 (2)	0.000	0.000
I2	0.0790 (3)	0.1007 (4)	0.1243 (4)	0.0253 (2)	−0.0064 (3)	0.0184 (3)

### Geometric parameters (Å, °)

**Table:**

C1—C2	1.368 (7)	C8—H8C	0.9600
C1—C5	1.375 (6)	C9—H9A	0.9600
C1—H1	0.9300	C9—H9B	0.9600
C2—N1	1.320 (7)	C9—H9C	0.9600
C2—H2	0.9300	C7'—H7'1	0.9600
C3—N1	1.327 (7)	C7'—H7'2	0.9600
C3—C4	1.374 (7)	C7'—H7'3	0.9600
C3—H3	0.9300	C8'—H8'1	0.9600
C4—C5	1.389 (6)	C8'—H8'2	0.9600
C4—H4	0.9300	C8'—H8'3	0.9600
C5—C6	1.528 (6)	C9'—H9'1	0.9600
C6—C8'	1.44 (2)	C9'—H9'2	0.9600
C6—C7	1.46 (2)	C9'—H9'3	0.9600
C6—C9"	1.50 (2)	C8"—H8"1	0.9600
C6—C9	1.500 (16)	C8"—H8"2	0.9600
C6—C7'	1.52 (3)	C8"—H8"3	0.9600
C6—C8"	1.549 (16)	C7"—H7"1	0.9600
C6—C9'	1.55 (2)	C7"—H7"2	0.9600
C6—C8	1.614 (19)	C7"—H7"3	0.9600
C6—C7"	1.641 (17)	C9"—H9"1	0.9600
C7—H7A	0.9600	C9"—H9"2	0.9600
C7—H7B	0.9600	C9"—H9"3	0.9600
C7—H7C	0.9600	N1—H99	0.90 (2)
C8—H8A	0.9600	I1—I2^i^	2.9105 (4)
C8—H8B	0.9600	I1—I2	2.9105 (4)
			
C2—C1—C5	120.2 (5)	H7A—C7—H7C	109.5
C2—C1—H1	119.9	H7B—C7—H7C	109.5
C5—C1—H1	119.9	C6—C8—H8A	109.5
N1—C2—C1	122.4 (5)	C6—C8—H8B	109.5
N1—C2—H2	118.8	H8A—C8—H8B	109.5
C1—C2—H2	118.8	C6—C8—H8C	109.5
N1—C3—C4	121.1 (5)	H8A—C8—H8C	109.5
N1—C3—H3	119.4	H8B—C8—H8C	109.5
C4—C3—H3	119.4	C6—C9—H9A	109.5
C3—C4—C5	120.5 (5)	C6—C9—H9B	109.5
C3—C4—H4	119.7	H9A—C9—H9B	109.5
C5—C4—H4	119.7	C6—C9—H9C	109.5
C1—C5—C4	116.5 (4)	H9A—C9—H9C	109.5
C1—C5—C6	122.8 (4)	H9B—C9—H9C	109.5
C4—C5—C6	120.7 (4)	C6—C7'—H7'1	109.5
C8'—C6—C9"	141.9 (13)	C6—C7'—H7'2	109.5
C7—C6—C9"	111.9 (14)	H7'1—C7'—H7'2	109.5
C8'—C6—C9	132.2 (12)	C6—C7'—H7'3	109.5
C7—C6—C9	127.3 (12)	H7'1—C7'—H7'3	109.5
C8'—C6—C7'	105.7 (14)	H7'2—C7'—H7'3	109.5
C9"—C6—C7'	64.7 (14)	C6—C8'—H8'1	109.5
C9—C6—C7'	85.2 (13)	C6—C8'—H8'2	109.5
C8'—C6—C5	108.8 (9)	H8'1—C8'—H8'2	109.5
C7—C6—C5	113.2 (10)	C6—C8'—H8'3	109.5
C9"—C6—C5	108.8 (9)	H8'1—C8'—H8'3	109.5
C9—C6—C5	109.3 (7)	H8'2—C8'—H8'3	109.5
C7'—C6—C5	112.3 (11)	C6—C9'—H9'1	109.5
C7—C6—C8"	78.9 (12)	C6—C9'—H9'2	109.5
C9"—C6—C8"	130.8 (11)	H9'1—C9'—H9'2	109.5
C9—C6—C8"	114.0 (11)	C6—C9'—H9'3	109.5
C7'—C6—C8"	123.5 (13)	H9'1—C9'—H9'3	109.5
C5—C6—C8"	109.8 (6)	H9'2—C9'—H9'3	109.5
C8'—C6—C9'	112.1 (14)	C6—C8"—H8"1	109.5
C7—C6—C9'	136.4 (12)	C6—C8"—H8"2	109.5
C7'—C6—C9'	107.7 (14)	H8"1—C8"—H8"2	109.5
C5—C6—C9'	110.2 (8)	C6—C8"—H8"3	109.5
C8"—C6—C9'	90.7 (11)	H8"1—C8"—H8"3	109.5
C7—C6—C8	104.9 (12)	H8"2—C8"—H8"3	109.5
C9"—C6—C8	109.8 (12)	C6—C7"—H7"1	109.5
C9—C6—C8	89.6 (11)	C6—C7"—H7"2	109.5
C7'—C6—C8	138.5 (13)	H7"1—C7"—H7"2	109.5
C5—C6—C8	108.2 (8)	C6—C7"—H7"3	109.5
C9'—C6—C8	64.5 (11)	H7"1—C7"—H7"3	109.5
C8'—C6—C7"	82.5 (11)	H7"2—C7"—H7"3	109.5
C9"—C6—C7"	90.8 (11)	C6—C9"—H9"1	109.5
C9—C6—C7"	110.2 (10)	C6—C9"—H9"2	109.5
C5—C6—C7"	109.6 (7)	H9"1—C9"—H9"2	109.5
C8"—C6—C7"	103.7 (10)	C6—C9"—H9"3	109.5
C9'—C6—C7"	129.5 (10)	H9"1—C9"—H9"3	109.5
C8—C6—C7"	127.6 (10)	H9"2—C9"—H9"3	109.5
C6—C7—H7A	109.5	C2—N1—C3	119.3 (4)
C6—C7—H7B	109.5	C2—N1—H99	120 (9)
H7A—C7—H7B	109.5	C3—N1—H99	121 (9)
C6—C7—H7C	109.5	I2^i^—I1—I2	177.55 (3)

Symmetry codes: (i) −*x*+1/2, −*y*+1/2, *z*.

### Hydrogen-bond geometry (Å, °)

**Table:**

*D*—H···*A*	*D*—H	H···*A*	*D*···*A*	*D*—H···*A*
N1—H99···N1^ii^	0.90	1.76	2.655 (7)	172

Symmetry codes: (ii) −*x*+1/2, −*y*+3/2, *z*.
